# Supplementary material for: The chromatin regulator Ankrd11 controls cardiac neural crest cell-mediated outflow tract remodeling and heart function
Source: Nat Commun. 2024 Jul 1;15:4632. doi: 10.1038/s41467-024-48955-1 (PMC11217281; doi:10.1038/s41467-024-48955-1)
Supplement: Supplementary file 1 — Supplementary Information [file 41467_2024_48955_MOESM1_ESM.pdf]

Supplemental data for

The chromatin regulator Ankrd11 controls cardiac neural crest cell-mediated outflow tract remodeling and heart function

Yana Kibalnyk[1,2], Elia Afanasiev[3], Ronan M. N. Noble[2,4], Adrianne ES Watson[1,2], Irina Poverennaya[5], Nicole L. Dittmann[1,6], Maria Alexiou[7], Kara Goodkey[1,2], Amanda A. Greenwell[2,8], John R. Ussher[2,8], Igor Adameyko[5,9], James Massey[10], Daniel Graf[1,2,7], Stephane L. Bourque[2,11], Jo Anne Stratton[3] and Anastassia Voronova[1-2, 6, 12*]

[1] Department of Medical Genetics, Faculty of Medicine & Dentistry, University of Alberta, Edmonton, Alberta, T6G 2H7, Canada

[2] Women and Children's Health Research Institute, 5-083 Edmonton Clinic Health Academy, University of Alberta, 11405 87 Avenue NW Edmonton, Alberta, Canada, T6G 1C9

[3] Department of Neurology and Neurosurgery, Montreal Neurological Institute, McGill University, Montreal, H3A 2B4, Canada

[4] Department of Pediatrics, Faculty of Medicine & Dentistry, University of Alberta, Edmonton, AB T6G 2G3, Canada.

[5] Department of Neuroimmunology, Center for Brain Research, Medical University of Vienna, 1090 Vienna, Austria

[6] Neuroscience and Mental Health Institute, Faculty of Medicine & Dentistry, University of Alberta, Edmonton, Alberta, T6G 2E1, Canada

[7] Department of Dentistry, Faculty of Medicine & Dentistry, University of Alberta, Edmonton, Alberta, T6G 2H7, Canada

[8] Faculty of Pharmacy & Pharmaceutical Sciences, University of Alberta, Edmonton, Alberta, Edmonton, Alberta, Canada T6G 2H1

[9] Department of Physiology and Pharmacology, Karolinska Institutet, 17177 Stockholm, Sweden

[10] Vizgen Inc., Cambridge, MA, USA

[11] Department of Anesthesiology & Pain Medicine, Faculty of Medicine & Dentistry, University of Alberta, Edmonton, Alberta, T6G 2G3, Canada

[12] Department of Cell Biology, Faculty of Medicine & Dentistry, University of Alberta, Edmonton, Alberta, T6G 2H7, Canada

*Corresponding author: 8-39 Medical Sciences Building, University of Alberta, Edmonton, Alberta, T6G 2H7, Canada. voronova@ualberta.ca

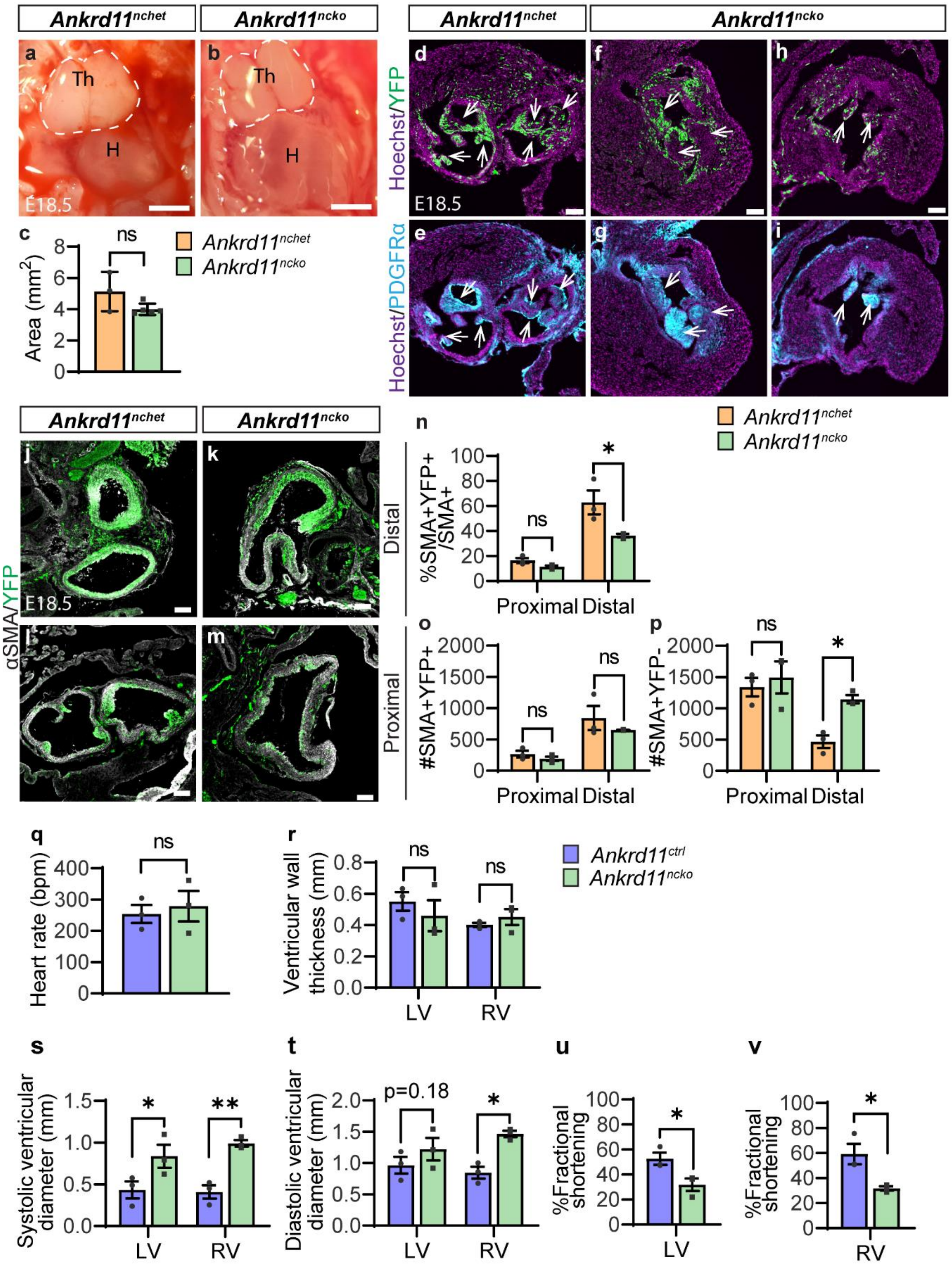

Ankrd11nchet
Ankrd11ncko
a
b
Th
H
E18.5
c
ns
Area (mm2)
0
2
4
6
8
Ankrd11nchet
Ankrd11ncko
d
e
f
g
h
i
Hoechst/YFP
Hoechst/PDGFRα
E18.5
j
k
l
m
αSMA/YFP
E18.5
Distal
Proximal
n
%SMA+YFP+/SMA+
0
20
40
60
80
100
ns
*
Proximal
Distal
o
#SMA+YFP+
0
500
1000
1500
2000
ns
ns
Proximal
Distal
p
#SMA+YFP-
0
500
1000
1500
2000
ns
*
Proximal
Distal
q
Heart rate (bpm)
0
100
200
300
400
ns
r
Ventricular wall thickness (mm)
0.0
0.2
0.4
0.6
0.8
ns
ns
LV
RV
Ankrd11ctrl
Ankrd11ncko
s
Systolic ventricular diameter (mm)
0.0
0.5
1.0
1.5
*
**
LV
RV
t
Diastolic ventricular diameter (mm)
0.0
0.5
1.0
1.5
2.0
p=0.18
*
LV
RV
u
%Fractional shortening
0
20
40
60
80
100
*
LV
v
%Fractional shortening
0
20
40
60
80
100
*
RV

Supplemental Figure 1. Conditional loss of *Ankrd11* in the neural crest causes increased ventricular diameter, decreased fractional shortening, and dysplastic valves at E18.5. a-b. Representative images of *Ankrd11*^{nchet} (a) and *Ankrd11*^{ncko} (b) thymus at E18.5. **c.** Quantification of a-b for total thymus area in *Ankrd11*^{nchet} (orange) and *Ankrd11*^{ncko} (green) genotypes. Mann-Whitney test. **d-i.** Representative images of *Ankrd11*^{nchet} (d, e) and *Ankrd11*^{ncko} (f-i) transverse sections of aortic and pulmonary (*Ankrd11*^{nchet}) or truncal (*Ankrd11*^{ncko}) valves (arrows) immunostained for CNCC derivatives (YFP; green; d, f-h), PDGFRα (blue; e, g-i) and counterstained for cell nuclei (Hoechst; magenta). **j-m.** Representative images of *Ankrd11*^{nchet} (j, l) and *Ankrd11*^{ncko} (k, m) transverse sections of distal (j-k) and proximal (l-m) aortic and pulmonary (*Ankrd11*^{nchet}) or truncal (*Ankrd11*^{ncko}) vessels immunostained for CNCC derivatives (YFP; green) and αSMA (white). **n-p.** Quantification of j-m for proportion of smooth muscle cells that are CNCC derived (% αSMA+YFP+/αSMA+; n), number of αSMA+YFP+ cells (o) and αSMA+YFP- cells (p) within the αSMA+ vessels. Two-tailed multiple t-tests with Holm-Sidak multiple comparisons test (n, distal p= 0.010763; p, distal p= 0.034171). **q-t.** Quantification of supplemental video files for fetal heart rate (q, bpm: beats per minute), diastolic ventricular wall thickness (r), systolic (s) and diastolic (t) diameter of the left (LV) and right ventricle (RV) of *Ankrd11*^{ctrl} (*Ankrd11*$^{fl/fl}$ or *Ankrd11*^{nchet}; blue bars) and *Ankrd11*^{ncko} (green bars) embryos. Two-tailed unpaired t-test (q), two-tailed multiple t-tests with Holm-Sidak multiple comparisons test (r-t; s, LV p= 0.018753, RV p= 0.005594, t, RV p= 0.015199). **u-v.** Quantification of percent fractional shortening (% change between the end diastolic and end systolic ventricular diameters) in LV (u) and RV (v). Two-tailed unpaired t-test (u, p= 0.0406; v, p= 0.0307). All ventricular measurements were taken from m-mode short axis images. ns: not significant, * $p < 0.05$ ** $p < 0.01$. Graphs represent mean ± s.e.m; n=3 *Ankrd11*^{nchet} and 5 *Ankrd11*^{ncko} (a-c); n=5 *Ankrd11*^{nchet} and 5 *Ankrd11*^{ncko} (d-i); n=3 *Ankrd11*^{nchet} and 3 *Ankrd11*^{ncko} (n-p); n=3 *Ankrd11*^{ctrl} and 3 *Ankrd11*^{ncko} (q-v). Embryos were taken from at least 2 independent litters. Scale bars: 1 mm (a-b), 100 µm (d-h, j-m). Source data are provided as a Source Data file.

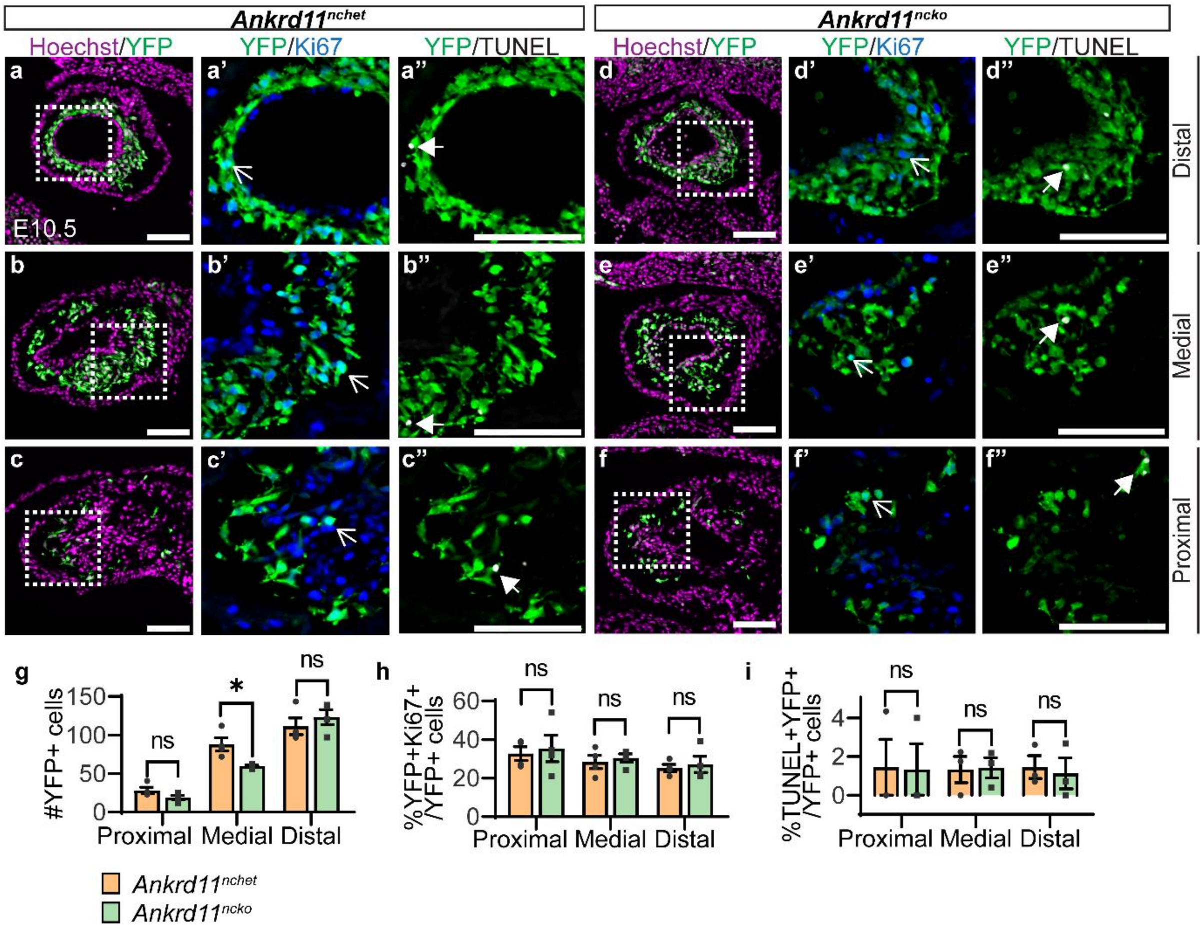

Supplemental Figure 2. Conditional loss of *Ankrd11* in the neural crest causes decreased CNCC number in OFT medial region at E10.5 without differences in proliferation or apoptosis. a-f. Representative images of *Ankrd11*^{nchet} (a-c") and *Ankrd11*^{ncko} (d-f") OFT at E10.5 divided into proximal, medial, and distal zones and immunostained for CNCC derivatives (YFP; green) and counterstained for cell nuclei (Hoechst; magenta). **a'-f''.** Magnified images of OFT regions (white dashed boxes in e-j) immunostained for Ki67 (blue), YFP (green) and TUNEL (white). Open arrowheads indicate examples of YFP+ Ki67+ CNCCs and closed arrowheads indicate examples of YFP+TUNEL+ CNCCs. **g-i.** Quantification of a-f for average number of YFP+ CNCC cells (g), proliferative index of CNCC cells (% YFP+Ki67+/YFP+; h) and proportion of apoptotic CNCC cells (% TUNEL+YFP+/YFP+; i) in proximal, medial, and distal OFT regions between *Ankrd11*^{nchet} (orange) and *Ankrd11*^{ncko} (green) genotypes at E10.5. Two-tailed multiple t-tests with Holm-Sidak multiple comparisons test (g, medial p= 0.038597). ns: not significant, * $p < 0.05$. Graphs represent mean ± s.e.m; n=4 *Ankrd11*^{nchet} and 4 *Ankrd11*^{ncko} (g-h); n=3 *Ankrd11*^{nchet} and 3 *Ankrd11*^{ncko} (i) from at least 2 independent litters. Scale bars: 100 µm. Source data are provided as a Source Data file.

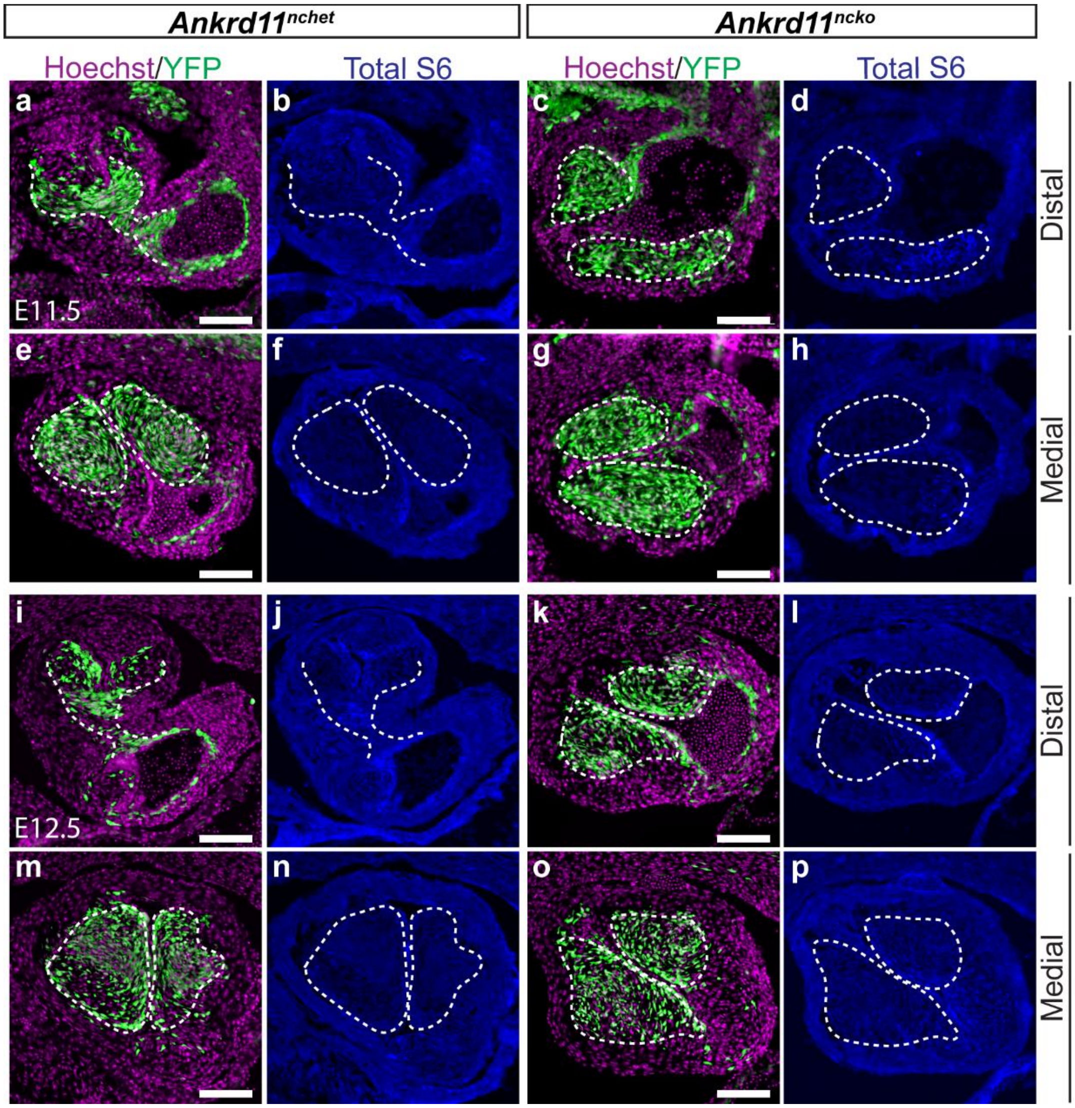

Supplemental Figure 3. Conditional loss of *Ankrd11* in the neural crest does not cause differences in total S6 protein levels. a-p. Representative images of *Ankrd11*^{nchet} (a-b, e-f, i-j, m-n) and *Ankrd11*^{ncko} (c-d, g-h, k-l, o-p) distal and medial OFT cushions (outlined with white dashed lines) at E11.5 (a-h) and E12.5 (i-p) immunostained for CNCCs (YFP; green), total S6 (blue) and counterstained for nuclei (Hoechst, magenta). YFP+ images also appear in Figure 5.

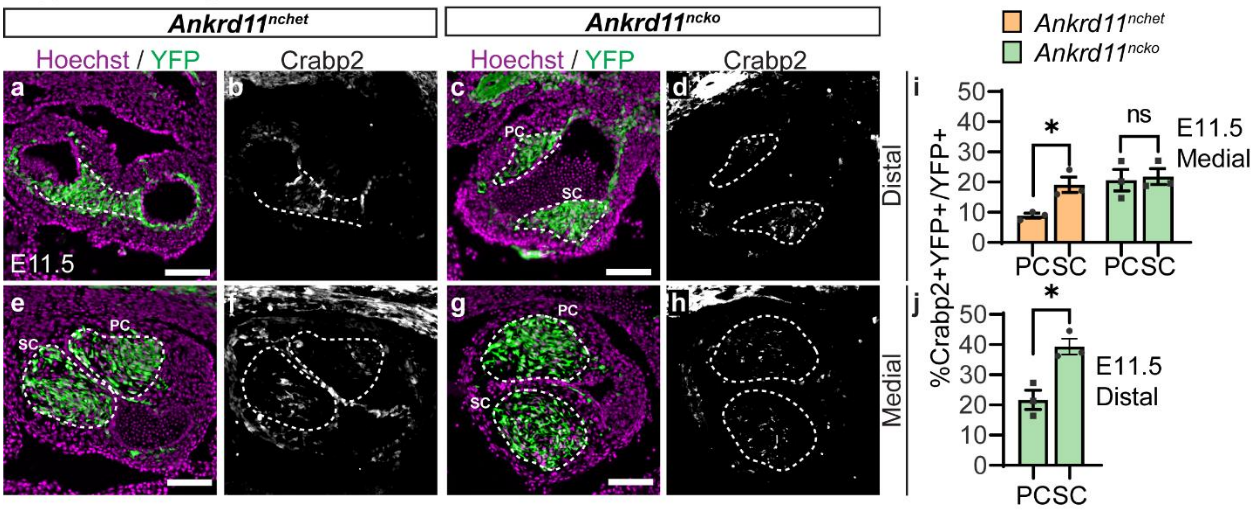

Supplemental Figure 4. Conditional loss of *Ankrd11* in the neural crest causes spatially impaired Crabp2 protein level asymmetry in OFT cushions at E11.5. a-h. Representative images of E11.5 *Ankrd11*^{nchet} (a-b, e-f) and *Ankrd11*^{ncko} (c-d, g-h) distal and medial OFT cushions (outlined with white dashed lines) immunostained for CNCCs (YFP; green), Crabp2 (white) and counterstained for nuclei (Hoechst, magenta). **i-j**. Quantification of a-h for percent of Crabp2+ CNCCs (%Crabp2+YFP+/YFP+ cells) in *Ankrd11*^{nchet} (orange) and *Ankrd11*^{ncko} medial (green) OFT (i) and in the *Ankrd11*^{ncko} distal OFT (j), at E11.5 in the parietal and septal cushions. Two-tailed multiple t-tests with Holm-Sidak multiple comparisons test (i, *Ankrd11*^{nchet} p= 0.046044); two-tailed unpaired t-test (j, 0.0126). Graphs represent mean ± s.e.m; n=3 *Ankrd11*^{nchet} and 3 *Ankrd11*^{ncko} from at least 2 independent litters. ns: not significant, * $p < 0.05$. Scale bars: 100 µm. Source data are provided as a Source Data file.

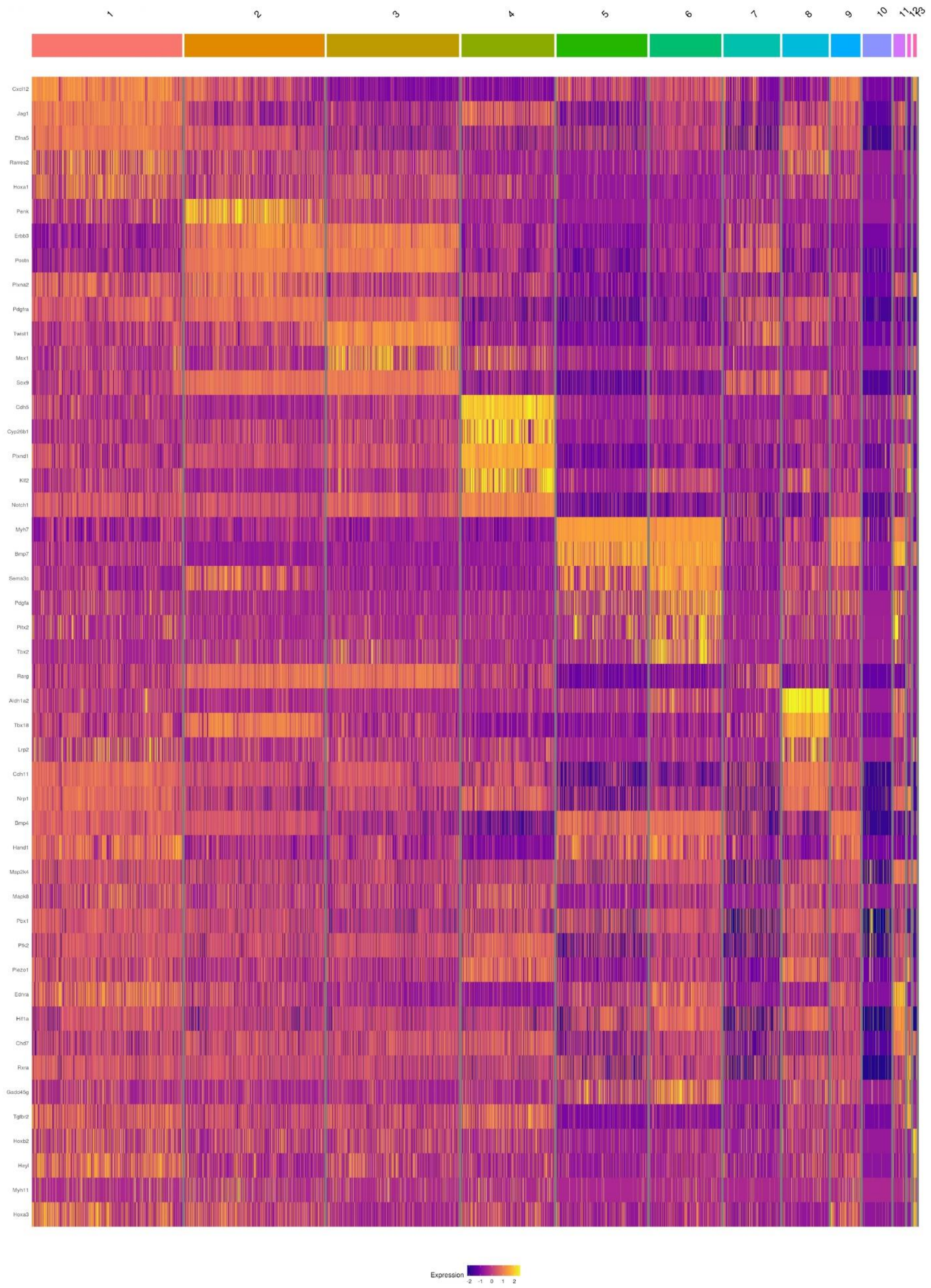
1
2
3
4
5
6
7
8
9
10
11
12
13
Cxcl12
Jag1
Efna5
Rarres2
Hoxa1
Penk
Erbb3
Postn
Plxna2
Pdgfra
Twist1
Msx1
Sox9
Cdh5
Cyp26b1
Plxnd1
Klf2
Notch1
Myh7
Bmp7
Sema3c
Pdgfa
Pitx2
Tbx2
Rarg
Aldh1a2
Tbx18
Lrp2
Cdh11
Nrp1
Bmp4
Hand1
Map2k4
Mapk8
Pbx1
Ptk2
Piezo1
Ednra
Hif1a
Chd7
Rxra
Gadd45g
Tgfbr2
Hoxb2
Heyl
Myh11
Hoxa3
Expression
-2 -1 0 1 2

Supplemental Figure 5. MERFISH identified 13 clusters in the *Ankrd11^{nchet}* and *Ankrd11^{ncko}* OFT at E11.5. Heatmap plot showing scaled expression of cluster marker genes in OFT cells from all samples. Cells are ordered according to their clustering. Source data are provided as a Source Data file.

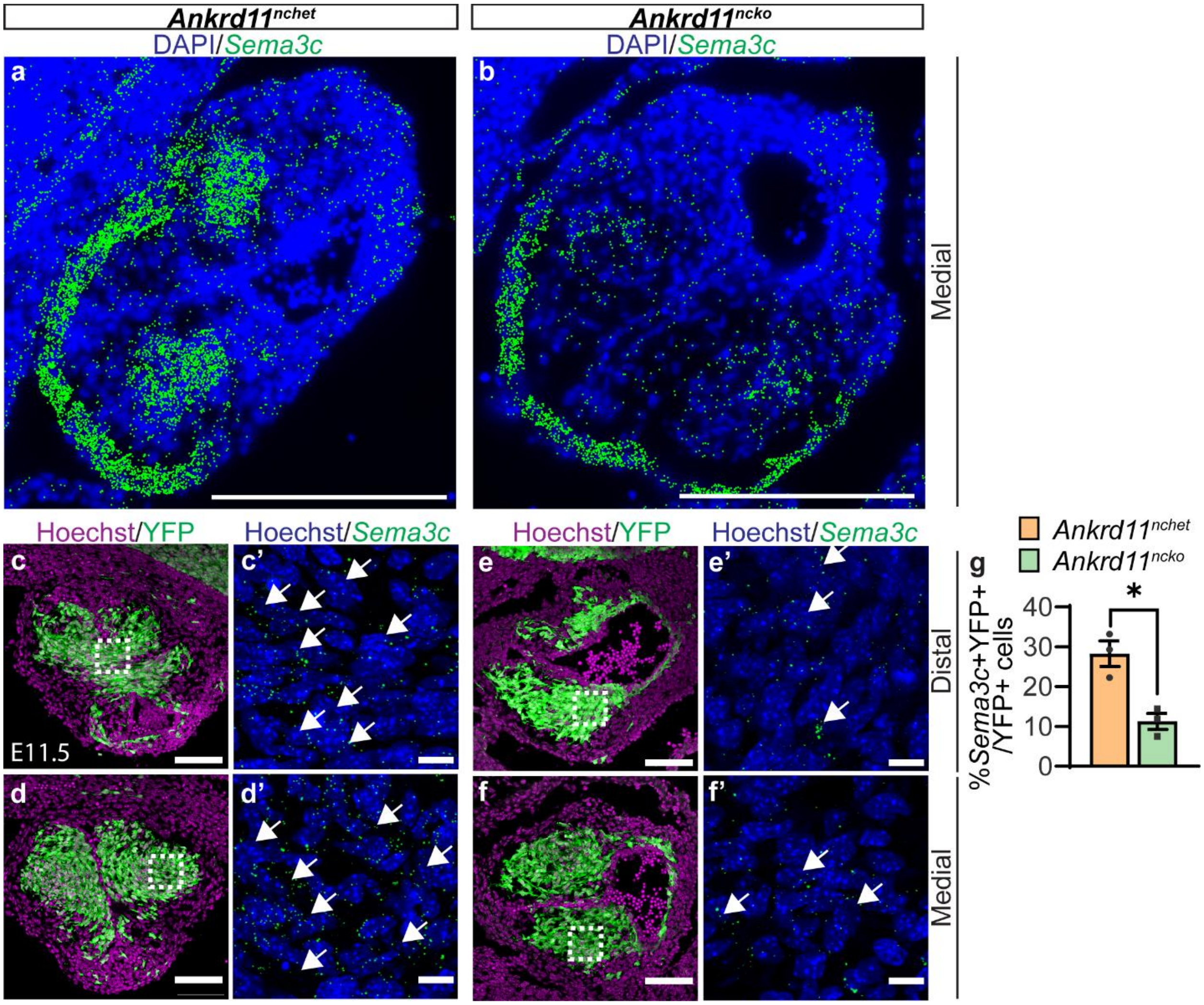

Supplemental Figure 6. Conditional loss of *Ankrd11* in the neural crest causes decreased *Sema3c* expression at E11.5. *Related to Figure 8.* **a-b.** Representative MERFISH images of *Ankrd11*^{nchet} (a) and *Ankrd11*^{ncko} (b) medial OFT sections displaying *Sema3c* probe signal (green) and nuclear signal (blue) (made with MERSCOPE Vizualizer). **c-f.** Representative images of *Ankrd11*^{nchet} (c-d) and *Ankrd11*^{ncko} (e-f) distal (c, e) and medial (d, f) OFT sections immunostained for CNCCs (YFP; green) and stained for nuclei (Hoechst, magenta). **c'-f'**. RNAscope magnified images of OFT regions (white dashed boxes in c-f) labeled with *Sema3c* mRNA probe (green) and counterstained for cell nuclei (Hoechst; blue). Arrows indicate examples *Sema3c*+ CNCCs. **g.** Quantification of *Sema3c*+YFP+ CNCCs in *Ankrd11*^{nchet} (orange) and *Ankrd11*^{ncko} (green) medial OFT cushions. Two-tailed unpaired t-test (p= 0.0108). Graph represents mean ± s.e.m; n=3 *Ankrd11*^{nchet} and 3 *Ankrd11*^{ncko} from at least 2 independent litters. * $p < 0.05$. Scale bars: 250 µm (a-b), 100 µm (c-f), 10 µm (c'-f'). Source data are provided as a Source Data file.
