# Supplementary material for: The chromatin regulator Ankrd11 controls cardiac neural crest cell-mediated outflow tract remodeling and heart function
Source: Nat Commun. 2024 Jul 1;15:4632. doi: 10.1038/s41467-024-48955-1 (PMC11217281; doi:10.1038/s41467-024-48955-1)
Supplement: Supplementary file 3 — Description of Additional Supplementary Files [file 41467_2024_48955_MOESM3_ESM.pdf]

Description of Additional Supplementary Files

File Name: Supplementary Data 1
Description: List of 140 genes in the MERFISH probe panel.

File Name: Supplementary Data 2
Description: List of differentially expressed genes between control and Ankrd11ncko in CNCCs, calculated using the Wilcoxon rank-sum test.

File Name: Supplementary Movie 1
Description: Representative long axis B-mode recordings of Ankrd11^{ctrl} *in-utero* echocardiography.

File Name: Supplementary Movie 2
Description: Representative long axis B-mode recordings of Ankrd11^{ncko} *in-utero* echocardiography.
