# Supplementary material for: The chromatin regulator Ankrd11 controls cardiac neural crest cell-mediated outflow tract remodeling and heart function
Source: Nat Commun. 2024 Jul 1;15:4632. doi: 10.1038/s41467-024-48955-1 (PMC11217281; doi:10.1038/s41467-024-48955-1)
Supplement: Supplementary file 8 — Reporting Summary [file 41467_2024_48955_MOESM8_ESM.pdf]

nature portfolio

Corresponding author(s): Anastassia Voronova

Last updated by author(s): Apr 23, 2024

Reporting Summary

Nature Portfolio wishes to improve the reproducibility of the work that we publish. This form provides structure for consistency and transparency in reporting. For further information on Nature Portfolio policies, see our Editorial Policies and the Editorial Policy Checklist.

Statistics

For all statistical analyses, confirm that the following items are present in the figure legend, table legend, main text, or Methods section.

n/a	Confirmed	
☐	☒	The exact sample size (*n*) for each experimental group/condition, given as a discrete number and unit of measurement
☐	☒	A statement on whether measurements were taken from distinct samples or whether the same sample was measured repeatedly
☐	☒	The statistical test(s) used AND whether they are one- or two-sided<br>*Only common tests should be described solely by name; describe more complex techniques in the Methods section.*
☒	☐	A description of all covariates tested
☐	☒	A description of any assumptions or corrections, such as tests of normality and adjustment for multiple comparisons
☐	☒	A full description of the statistical parameters including central tendency (e.g. means) or other basic estimates (e.g. regression coefficient) AND variation (e.g. standard deviation) or associated estimates of uncertainty (e.g. confidence intervals)
☐	☒	For null hypothesis testing, the test statistic (e.g. *F*, *t*, *r*) with confidence intervals, effect sizes, degrees of freedom and *P* value noted<br>*Give P values as exact values whenever suitable.*
☒	☐	For Bayesian analysis, information on the choice of priors and Markov chain Monte Carlo settings
☒	☐	For hierarchical and complex designs, identification of the appropriate level for tests and full reporting of outcomes
☒	☐	Estimates of effect sizes (e.g. Cohen's *d*, Pearson's *r*), indicating how they were calculated

Our web collection on statistics for biologists contains articles on many of the points above.

Software and code

Policy information about availability of computer code

Data collection	All fluorescence image acquisition was performed using Zen software (Zeiss). MicroCT was performed with the Milabs UHT-µCT system. Echocardiography was performed with the Vevo 3100 system. MERFISH imaging was performed on the MERSCOPE system (Vizgen 10000001).
Data analysis	MicroCT images were analyzed using 3D Slicer (version 5.0.3). All images except for MERFISH were counted, analyzed, and representative images were processed in Fiji ImageJ v1.53t software. Statistical analysis was performed with Prism (version 8.0.2). Figures were generated in Adobe Illustrator CC 2015. Single cell RNA sequencing data re-analysis was done using scanpy (version 1.9.1), seaborn (version 0.12.1), and pandas v1.5.2. MERFISH analysis was done using MERlin v0.1.6, Cellpose, squidpy (1.3.1, python version 3.11.6), Seurat (5.0.1, R version 4.3.3), MERSCOPE Vizualizer Version: 2.3.3330.0. The MERFISH analysis source code as well as processed and meta- data is available in GitHub [https://doi.org/10.5281/zenodo.10998925].

For manuscripts utilizing custom algorithms or software that are central to the research but not yet described in published literature, software must be made available to editors and reviewers. We strongly encourage code deposition in a community repository (e.g. GitHub). See the Nature Portfolio guidelines for submitting code & software for further information.

Data

Policy information about availability of data

All manuscripts must include a data availability statement. This statement should provide the following information, where applicable:

- Accession codes, unique identifiers, or web links for publicly available datasets
- A description of any restrictions on data availability
- For clinical datasets or third party data, please ensure that the statement adheres to our policy

The scRNAseq data from Soldatov et al. 2019 used in this study are available in the GEO database under accession code GSE129114 [https://www.ncbi.nlm.nih.gov/geo/query/acc.cgi?acc=GSE129114].
The MERFISH post segmentation cell/gene expression count matrices and associated per-cell metadata generated in this study have been deposited in the GEO database under accession code GSE258835 [https://www.ncbi.nlm.nih.gov/geo/query/acc.cgi?acc=GSE258835]. This integrated object has also been made available on our open science website: SingloCell [singlocell.openscience.mcgill.ca/NeuralCrestAnkrd11-KO] (AH59.5 = ncHET 1; AH59.6 = ncHET 2; AIO4.2 = ncHET 3; AH50.1 = ncKO 1; AH59.2 = ncKO 2; AI13.3 = ncKO 3)
Source data are provided with this paper.

Research involving human participants, their data, or biological material

Policy information about studies with human participants or human data. See also policy information about sex, gender (identity/presentation), and sexual orientation and race, ethnicity and racism.

Reporting on sex and gender	N/A
Reporting on race, ethnicity, or other socially relevant groupings	N/A
Population characteristics	N/A
Recruitment	N/A
Ethics oversight	N/A

Note that full information on the approval of the study protocol must also be provided in the manuscript.

Field-specific reporting

Please select the one below that is the best fit for your research. If you are not sure, read the appropriate sections before making your selection.

☒ Life sciences ☐ Behavioural & social sciences ☐ Ecological, evolutionary & environmental sciences

For a reference copy of the document with all sections, see nature.com/documents/nr-reporting-summary-flat.pdf

Life sciences study design

All studies must disclose on these points even when the disclosure is negative.

Sample size	Sample size was not predetermined through statistical tests. A minimum n=3 per genotype from 2 independent litters was used to calculate statistical significance. Sample sizes are consistent with those used in a previous publication (PMID: 33996804). N=3-8 per genotype was used per experiment.
Data exclusions	In Fig. 2f, one Ankrd11ctrl data point and in Fig. 2g one Ankrd11ncko point was excluded using the ROUT method with Q=1% to identify outliers.
Replication	Results were presented from at least 3 embryos from each genotype, taken from at least 2 independent litters. All replication attempts were successful.
Randomization	No randomization was performed, as embryos were allocated to experiments by age and genotype. Covariates were not relevant to the study due to the full penetrance of the OFT phenotype regardless of embryo sex or parent ID.
Blinding	No investigator blinding methods were used during analysis due to the visibly obvious phenotype of Ankrd11ncko samples. Dams were randomly assigned to each experiment.

Reporting for specific materials, systems and methods

We require information from authors about some types of materials, experimental systems and methods used in many studies. Here, indicate whether each material, system or method listed is relevant to your study. If you are not sure if a list item applies to your research, read the appropriate section before selecting a response.

Materials & experimental systems

n/a	Involved in the study
☐	☒ Antibodies
☒	☐ Eukaryotic cell lines
☒	☐ Palaeontology and archaeology
☐	☒ Animals and other organisms
☒	☐ Clinical data
☒	☐ Dual use research of concern
☒	☐ Plants

Methods

n/a	Involved in the study
☒	☐ ChIP-seq
☒	☐ Flow cytometry
☒	☐ MRI-based neuroimaging

Antibodies

Antibodies used

Primary antibodies and stains: anti-Crabp2 (Proteintech, 10225-1-AP, Lot 00051203, 1:200), anti-eGFP (Abcam, ab13970, Lot GR3361051-16, 1:2000), anti-Ki67 (BD Pharmingen, 556003, Lot 1119219, 1:500), anti-αSMA (Sigma, A2547, Lot 099M4848V, 1:1000), anti-PDGFRα (R&D Systems, AF1062, Lot HMQ0220101, 1:500), anti-pSmad1/5/8 (Cell Signalling, 13820, Lot 4, 1:300), anti-pSmad2/3 (Cell Signalling, 8828, Lot 8, 1:300), anti-S6 (Cell Signalling, 2317, Lot 13, 1:600), anti-pS6 (phosphoSer240/244; Cell Signalling, 2215, Lot 18, 1:600), biotinylated IB4 (Vector Laboratories, VECTB1205, Lot ZB1017, 1:1000), Phalloidin-iFluor647 (Abcam, ab176759, Lot GR3279773-9, 1:1000).
Secondary antibodies: anti-chicken-Alexa488 (Jackson, 703-545-155, Lot 162189, 1:1000), anti-chicken-Alexa647 (Jackson, 703-605-155, Lot 153969, 1:1000), anti-goat-Alexa647 (Jackson, 705-605-147, Lot 154191, 1:1000), anti-rabbit-Alexa647 (Jackson, 711-605-152, Lot 154880, 1:1000), streptavidin-Cy3 (Jackson, 016-160-084, Lot 168280(2), 1:1000), streptavidin-Cy5 (Jackson, 016-170-084, Lot 151872, 1:1000).

Validation

The antibodies have been verified by the manufacturer for IF or IHC. Validation is available on their website.
anti-Crabp2 https://www.ptglab.com/products/CRABP2-Antibody-10225-1-AP.htm
anti-eGFP https://www.abcam.com/products/primary-antibodies/gfp-antibody-ab13970.html
anti-Ki67 https://www.bdbiosciences.com/en-ca/products/reagents/microscopy-imaging-reagents/immunofluorescence-reagents/purified-mouse-anti-ki-67.556003
anti-αSMA https://www.sigmaaldrich.com/CA/en/product/sigma/a2547
anti-PDGFRα https://www.rndsystems.com/products/mouse-pdgf-ralpha-antibody_af1062
anti-pSmad1/5/8 https://www.cellsignal.com/products/primary-antibodies/phospho-smad1-ser463-465-smad5-ser463-465-smad9-ser465-467-d5b10-rabbit-mab/13820
anti-pSmad2/3 https://www.cellsignal.com/products/primary-antibodies/phospho-smad2-ser465-467-smad3-ser423-425-d27f4-rabbit-mab/8828
anti-S6 https://www.cellsignal.com/products/primary-antibodies/s6-ribosomal-protein-54d2-mouse-mab/2317
anti-pS6 https://www.cellsignal.com/products/primary-antibodies/phospho-s6-ribosomal-protein-ser240-244-antibody/2215
anti-chicken-Alexa488 https://www.jacksonimmuno.com/catalog/products/703-545-155
anti-chicken-Alexa647 https://www.jacksonimmuno.com/catalog/products/703-605-155
anti-goat-Alexa647 https://www.jacksonimmuno.com/catalog/products/705-605-147
anti-rabbit-Alexa647 https://www.jacksonimmuno.com/catalog/products/711-605-152

Animals and other research organisms

Policy information about studies involving animals; ARRIVE guidelines recommended for reporting animal research, and Sex and Gender in Research

Laboratory animals

All animal use was approved by the Animal Care Committee of the University of Alberta in accordance with the Canadian Council of Animal Care policies. All mice were housed in a University of Alberta Animal Facility and serviced by Health Sciences Laboratory Animal Services (HSLAS). Mice were maintained on a 14/10 hour light/dark cycle, at 21-23°C room temperature, and 40-70% humidity. Food and water was provided ad libitum. Embryos at developmental ages between E10.5-18.5 and of either sex were used for all experiments.
Ankrd11fl/fl mice, where exon 7 of the Ankrd11 gene was flanked by LoxP sites, were derived from the Ankrd11TM1a(EUCOMM)Wtsi//IcsOrl (Ankrd11tm1a) sperm (EM:07651, the European Mouse Mutant Archive – Infrafrontier) Ankrd11fl/fl mice were bred to hemizygous Wnt1Cre2+ mice (B6.Cg-E2f1Tg(Wnt1-cre)2Sor/J, stock # 022501, Jackson Laboratories), to create the Ankrd11fl/fl;Wnt1Cre2 mice. These were used for µCT analysis. Neural crest cell lineage tracing was performed by breeding in the RosaYFPSTOP allele using the RosaYFPSTOP/STOP mice (B6.129X1-Gt(ROSA)26Sortm1(EYFP)Cos/J, stock # 006148, Jackson Laboratories). These mice were used for all other analysis. The mouse line was maintained by crossing Ankrd11WT/WT;RosaYFPSTOP/STOP;WntCre2 with Ankrd11fl/fl;RosaYFPSTOP/STOP mice. Resulting Ankrd11fl/WT;RosaYFPSTOP/STOP;WntCre2 were timed mated with Ankrd11fl/fl;RosaYFPSTOP/STOP or Ankrd11fl/WT;RosaYFPSTOP/STOP mice to generate Ankrd11WT/WT;RosaYFPSTOP/STOP;Wnt1Cre2+ (Ankrd11WT/WT), Ankrd11fl/fl;RosaYFPSTOP/STOP and Ankrd11fl/WT;RosaYFPSTOP/STOP (Ankrd11ctrl), Ankrd11fl/WT;RosaYFPSTOP/STOP;Wnt1Cre2 (Ankrd11nchet) as well as Ankrd11fl/fl;RosaYFPSTOP/STOP;Wnt1Cre2 (Ankrd11ncko). For timed matings, mice were used at P40-100. Mice were mated in the evenings and a plug was determined in the morning, with positive plug considered as E0.25. For experiments, embryos were collected E10.5-E12.5 and E18.5.

Wild animals	The study did not involve wild animals.
Reporting on sex	Due to the full penetrance of the phenotype regardless of sex of the embryo, sex-based analysis was not performed.
Field-collected samples	The study did not involve samples collected from the field.
Ethics oversight	All animal use was approved by the Animal Care Committee of the University of Alberta in accordance with the Canadian Council of Animal Care policies.

Note that full information on the approval of the study protocol must also be provided in the manuscript.

Plants

Seed stocks	N/A
Novel plant genotypes	N/A
Authentication	N/A
